# Revision-Free Survival After MUTARS Total Knee Reconstruction in Limb Salvage Surgery: Primary Implantation Versus Conversion

**DOI:** 10.3390/cancers18091408

**Published:** 2026-04-29

**Authors:** Fabian Hille, Jan Christoph Theil, Georg Gosheger, Tymoteusz Budny, Marieke de Vaal, Anna Maria Rachbauer, Niklas Deventer

**Affiliations:** Department of Orthopedics and Tumororthopedics, University Hospital Münster, Albert-Schweitzer-Campus 1, 48149 Muenster, Germany; fhille@uni-muenster.de (F.H.); c.theil@orthopaedie-prinzipalmarkt.de (J.C.T.); tymoteusz.budny@ukmuenster.de (T.B.); mariekemathilda.devaal@ukmuenster.de (M.d.V.); anna.rachbauer@ukmuenster.de (A.M.R.)

**Keywords:** MUTARS Total Knee, megaprosthesis, limb salvage surgery

## Abstract

(1) **Background**: Megaprosthetic reconstruction of the knee is frequently required in limb salvage surgery for oncologic indications and in complex revision arthroplasty. The Modular Universal Tumor and Revision System (MUTARS) Total Knee prosthesis is widely used in these situations. The aim of this study was to evaluate revision-free implant survival following MUTARS Total Knee implantation and to compare outcomes between primary implantation and use as a conversion procedure after failure of a previous knee prosthesis. (2) **Methods**: A retrospective cohort study was performed including 36 patients who underwent MUTARS Total Knee implantation at a single institution. Patients were stratified into primary implantation (*n* = 24) and conversion after failed prosthesis (*n* = 12). The primary endpoint was time to first revision. (3) **Results**: Overall revision-free survival was 51.6% at 2 years and 29.3% at 5 years. No significant difference in revision-free survival was observed between primary implantation and conversion procedures (log-rank *p* = 0.67). When mechanical failure (Henderson type III) was considered as the sole endpoint, implant survival was substantially higher, with 88.3% survival at 2 years and 57.2% at 5 years. Infection-related implant survival was 72.0% at both 2 and 5 years. Secondary amputation was required in 7 of 36 patients (19.4%), with a higher proportion observed in the primary implantation group (25.0% vs. 8.3%). Most amputations occurred within the early postoperative period. (4) **Conclusions**: MUTARS Total Knee implantation is associated with a substantial revision burden; however, conversion after failed prosthesis did not result in inferior revision-free survival compared with primary implantation. Endpoint-specific analyses demonstrate that biological complications, particularly infection, represent the dominant drivers of early failure.

## Simple Summary

Megaprosthetic reconstruction of the knee is often required in limb salvage surgery for patients with bone tumors and in complex revision arthroplasty with severe bone loss. The MUTARS Total Knee system is commonly used in these challenging situations, but implant survival and complication patterns remain important concerns. In this study, we compared outcomes after primary implantation and use as a conversion procedure following failure of a previous knee prosthesis. While overall revision rates were high, implant survival did not differ significantly between groups. Importantly, a considerable proportion of patients required secondary amputation, most of which occurred early after surgery. These findings highlight the clinical complexity of limb salvage procedures and emphasize the role of biological complications, particularly infection, in treatment failure.

## 1. Introduction

Reconstruction of extensive knee defects remains one of the most challenging problems in contemporary knee arthroplasty. Large osseous and soft-tissue deficiencies may result from primary bone tumors, tumor-like lesions, periprosthetic fractures, severe bone loss after failed arthroplasty, or complex revision scenarios complicated by infection or instability [1,2,3]. In particular, malignant bone tumors of the lower extremity frequently require wide surgical resection to achieve local tumor control, often resulting in extensive segmental defects of the distal femur or proximal tibia involving the knee joint [4,5]. In these situations, limb salvage procedures must provide both mechanical stability and functional restoration while addressing massive bone loss [6,7,8].

Over the past decades, modular megaprosthetic replacement has become an established limb salvage strategy following sarcoma resection around the knee [9,10,11]. These implants allow immediate reconstruction of large osteoarticular defects and enable early mobilization compared with biological reconstruction techniques [10,12]. At the same time, megaprosthetic systems have increasingly been adopted in complex non-oncologic revision arthroplasty, particularly in cases of catastrophic bone loss or severe periprosthetic fractures where conventional revision total knee arthroplasty techniques reach their limits [13,14].

Among these systems, the Modular Universal Tumor and Revision System (MUTARS^®^) has gained wide acceptance due to its modularity, intraoperative flexibility, and applicability in both oncologic and non-oncologic settings [5,12]. The MUTARS^®^ Total Knee prosthesis is a modular megaprosthetic implant incorporating a rotating hinge mechanism and interchangeable segmental components, allowing intraoperative adaptation to variable resection lengths and defect configurations [12,15].

Despite their growing use, megaprosthetic knee reconstructions are associated with substantial complication rates [16,17,18]. Periprosthetic infection, mechanical or structural failure, aseptic loosening, and soft-tissue complications remain major challenges and frequently result in repeated revisions or even limb loss [16,17]. Reported implant survival rates vary considerably, reflecting heterogeneity in patient populations, surgical indications, and follow-up durations [19,20]. Infection in particular has repeatedly been identified as one of the most devastating complications in large segment reconstructions [17,21], while structural and soft-tissue failures also represent leading causes of revision in tumor endoprostheses [16,19].

Failure mechanisms in megaprosthetic reconstruction differ from those observed in conventional arthroplasty and are commonly classified according to the Henderson system, including soft-tissue failure, aseptic loosening, structural complications, infection, and tumor progression [16]. The application of standardized failure classification systems facilitates comparison across studies and underscores the complexity of survival analyses in this patient population [15].

The distinction between primary implantation and conversion after failed arthroplasty is clinically relevant but remains insufficiently explored in the literature [11,19]. Patients undergoing primary MUTARS^®^ Total Knee implantation—most commonly for oncologic indications—often differ substantially from those receiving the implant as a salvage solution after failed conventional arthroplasty [11,17]. Conversion cases are frequently characterized by compromised soft tissues, multiple prior surgical interventions, occult or treated infections, and altered biomechanics, all of which may adversely affect implant survival [13,22]. Conversely, primary implantations are typically performed in a more controlled surgical setting, albeit often following extensive oncologic resections [5,17]. Existing studies frequently combine primary and revision cases, potentially obscuring outcome differences between these fundamentally distinct indications [11,19]. Moreover, many reports focus predominantly on oncologic or mechanical outcomes, with comparatively limited emphasis on time-to-revision analyses reflecting the true clinical burden associated with these implants [17,22].

The purpose of the present study was therefore to evaluate revision-free implant survival following MUTARS^®^ Total Knee implantation, with a specific focus on comparing primary implantation to use of the system as a conversion procedure after failure of a previous knee prosthesis. Using time-to-event analysis, we sought to assess differences in revision-free survival between these two groups and to identify potential risk factors for revision. We hypothesized that MUTARS^®^ Total Knee implants used in conversion scenarios would demonstrate reduced revision-free survival compared with primary implantations.

## 2. Materials and Methods

### 2.1. Study Design

This retrospective cohort study evaluated revision-free implant survival following reconstruction of extensive knee defects using the Modular Universal Tumor and Revision System (MUTARS^®^) Total Knee prosthesis (Implantcast, Buxtehude, Germany). Ethical approval for retrospective data analysis was obtained from the local institutional review board (Ethics committee of the Westphalia-Lippe Medical Association and the Westphalian Wilhelms University of Münster, approval no. 2021-205-f-S, 21 December 2021), and all patients provided written informed consent.

All consecutive patients who underwent implantation of a MUTARS^®^ Total Knee prosthesis at our institution during the study period were screened for eligibility. Patients were included if complete follow-up data regarding revision status and time to revision were available. Both oncologic and non-oncologic indications were considered. Patients with incomplete follow-up or missing data on revision outcomes were excluded.

Patients were stratified into two groups based on the indication for implantation:

(1) Primary MUTARS^®^ Total Knee implantation, defined as first-time implantation without a previously implanted knee prosthesis;
(2) Conversion procedures, defined as implantation following failure of a prior knee prosthesis, including conventional total knee arthroplasty or other reconstructive procedures.

### 2.2. Surgical Treatment

All procedures were performed by experienced orthopedic surgeons specialized in complex knee reconstruction and musculoskeletal oncology. Surgical approaches and reconstruction strategies were individualized according to the underlying diagnosis, extent of bone and soft-tissue loss, and patient-specific factors.

**Figure 1:**
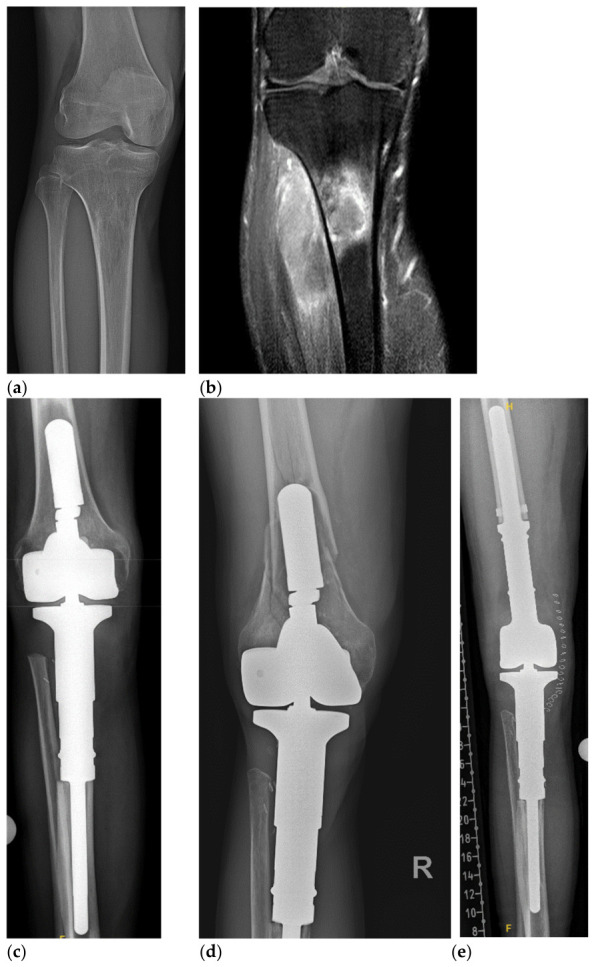
48 year old patient with a Ewing sarcoma of the proximal tibia: (**a**) radiography of the proximal tibia at initial diagnosis, (**b**) MRI of the proximal tibia at initial diagnosis, (**c**) radiography after tumor resection and reconstruction with a proximal tibia replacement, (**d**) radiography of the knee joint with periprosthetic fracture of the distal femur, and (**e**) radiography after conversion to a total knee replacement.

The MUTARS^®^ Total Knee system (Figure 1) was used in all cases. Its modular design allowed intraoperative adjustment of resection length, fixation technique, and joint configuration. Stem fixation was performed using cemented, uncemented, or hybrid techniques, depending on bone quality and surgeon preference. Additional reconstructive measures, including muscle flap coverage, attachment tubes, and the use of silver-coated components, were applied as clinically indicated to optimize soft-tissue management and potentially reduce infection risk.

Postoperative care followed standardized institutional protocols, including perioperative antibiotic prophylaxis, thromboprophylaxis, and individualized rehabilitation programs.

### 2.3. Henderson Classification

The primary endpoint was time to first revision, defined as the interval between implantation and the first revision surgery for any cause. Revision was defined as any surgical procedure involving exchange or removal of one or more prosthetic components, corresponding to Henderson failure types I–IV. Patients without revision were censored at the time of last clinical follow-up. Death without prior revision was treated as a censoring event.

Secondary outcomes included the distribution of failure modes according to the Henderson classification and the identification of potential risk factors associated with revision.

### 2.4. Statistical Analysis

Descriptive statistics were used to summarize patient demographics, clinical characteristics, and surgical variables. Continuous variables were reported as mean ± standard deviation or median with interquartile range, as appropriate. Categorical variables were presented as absolute numbers and percentages.

Revision-free implant survival was assessed using Kaplan–Meier analysis for the overall cohort and stratified by implantation indication. Survival rates at predefined time points were calculated. Differences between groups were evaluated using the log-rank test.

To identify potential independent predictors of revision, a Cox proportional hazards regression model was constructed. Covariates included patient age, implantation indication (primary versus conversion), use of silver-coated components, and radiotherapy exposure. Given the limited sample size, the number of covariates was restricted to reduce the risk of model overfitting. A Firth-corrected Cox proportional hazards regression was additionally performed as a sensitivity analysis to reduce potential small-sample bias. Results were reported as hazard ratios (HRs) with 95% confidence intervals (CIs).

All statistical analyses were performed using IBM SPSS Statistics Version 26 (IBM Corp., Armonk, NY, USA). A two-sided *p*-value of <0.05 was considered statistically significant.

## 3. Results

A total of 36 patients who underwent MUTARS^®^ Total Knee implantation were included in the analysis. Of these, 24 patients (67%) underwent primary implantation, whereas 12 patients (33%) received the implant as a conversion procedure following failure of a previous knee prosthesis. Complete follow-up data regarding revision status and time to revision were available for all patients included in the study.

Baseline demographic and surgical characteristics are summarized in Table 1. The mean age at implantation for the entire cohort was 46.3 ± 19.8 years, with comparable age distributions between the primary and conversion groups (48.5 ± 21.9 vs. 42.1 ± 14.8 years, respectively). Male patients accounted for 61.1% of the cohort. The median follow-up duration for the overall cohort was 44 months (interquartile range [IQR], 10–114 months), with a maximum follow-up of 193 months. Notably, follow-up duration differed between groups, with a longer median follow-up in the primary implantation group compared with the conversion group (55 vs. 28 months, respectively).

With regard to treatment-related variables, radiotherapy was administered exclusively in the primary implantation group (20.8%), reflecting the predominance of oncologic indications in this subgroup. Silver-coated prostheses were used in 58.3% of all cases, with a markedly higher proportion in the primary group (83.3%) compared with the conversion group (8.3%). Muscle flap reconstruction was performed in 61.1% of patients and was similarly distributed between groups. Cemented fixation was used in 41.7% of cases.

During the observation period, 24 patients (67%) experienced at least one revision procedure, whereas 12 patients (33%) remained free from revision and were censored at the time of last follow-up. Death without prior revision occurred in a subset of patients and was treated as a censoring event.

Kaplan–Meier analysis demonstrated a continuous decline in revision-free implant survival over time. For the entire cohort, revision-free survival was approximately 70% at 12 months, 52% at 24 months, and 29% at 60 months following implantation (Table 2).

**Figure 2:**
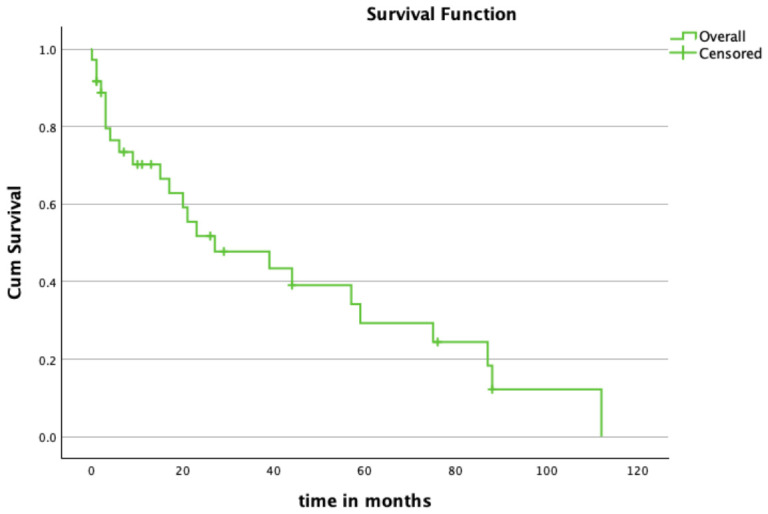
Overall Revision-Free Survival: Kaplan–Meier curve demonstrating revision-free implant survival for the overall cohort following MUTARS Total Knee implantation. The endpoint was first revision for any cause. Crosses indicate censored observations, defined as patients without revision at last follow-up or death without prior revision.

The median revision-free survival time was 27 months, defined as the time point at which the survival probability fell below 50% (Figure 2).

**Figure 3:**
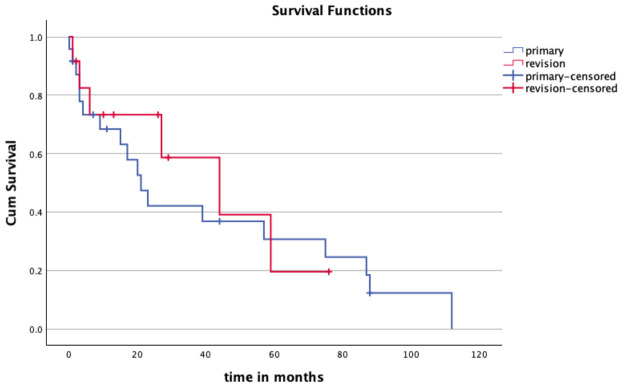
Kaplan–Meier curves comparing revision-free implant survival between primary MUTARS Total Knee implantation and MUTARS Total Knee implantation as a conversion procedure after failure of a previous knee prosthesis. The endpoint was first revision for any cause. Crosses indicate censored observations.

When stratified according to implantation indication, both groups exhibited substantial revision rates. In the primary implantation group, revision events tended to occur earlier, particularly within the first two years following implantation. In contrast, the conversion group demonstrated a more gradual decline in revision-free survival over time. However, despite these visual differences, the Kaplan–Meier curves showed considerable overlap, and no statistically significant difference in revision-free survival between groups was observed (log-rank test, *p* = 0.67) (Figure 3).

Failure modes according to the Henderson classification are presented in Table 3. Structural failure (type III) represented the most frequent cause of revision, accounting for 30.6% of cases. The most frequent failure mode was component fracture (*n* = 7), followed by periprosthetic fracture (*n* = 6) and mechanical loosening (*n* = 4), whereas component disconnection was observed in one case. This was followed by periprosthetic infection (type IV), which occurred in 22.2% of patients, and aseptic loosening (type II), observed in 11.1% of cases. Soft-tissue failure (type I) was rare, occurring in only one patient (2.8%). No cases of tumor progression (type V) were observed during the study period. The distribution of failure modes was broadly comparable between primary implantation and conversion procedures, with no distinct failure pattern specific to either group. When failure modes were analyzed separately as endpoints, survival estimates varied depending on the definition of implant failure. When structural failure (Henderson type III) was considered as the sole endpoint, revision-free survival was 88.3% at 2 years and 57.2% at 5 years. When infection (Henderson type IV) was analyzed as the sole endpoint, implant survival was 72.0% at both 2 and 5 years. These findings illustrate the substantial impact of the endpoint definition on survival estimates in megaprosthetic reconstruction.

Multivariable Cox proportional hazards regression analysis was performed to identify potential predictors of revision (Table 4). Covariates included age, implantation indication (primary versus conversion), obesity, smoking status, use of silver-coated components, radiotherapy exposure, and history of prior infection. None of the included variables demonstrated a statistically significant association with revision risk. Firth-corrected Cox regression yielded results comparable to the conventional Cox model and did not identify any statistically significant predictors of revision. Implantation as a conversion procedure was not associated with an increased risk of revision compared with primary implantation (hazard ratio [HR] 0.95; 95% confidence interval [CI] 0.10–8.76; *p* = 0.96). Radiotherapy exposure showed a nonsignificant trend toward increased risk (HR 1.79; 95% CI 0.29–11.19; *p* = 0.49). The wide confidence intervals observed for several variables reflect the limited sample size and should be interpreted with caution (Table 4).

A total of 7 of 36 patients (19.4%) underwent secondary amputation during follow-up. Amputation occurred in 6 of 24 patients (25.0%) in the primary MUTARS Total Knee group and in 1 of 12 patients (8.3%) in the conversion group. The median time to amputation for the overall cohort was 5 months. In the primary implantation group, amputations occurred after 1, 3, 5, 9, 24, and 82 months, whereas the single amputation in the conversion group occurred after 5 months. The underlying causes of secondary amputation were analyzed in detail. Persistent infection was the most frequent reason (*n* = 4), followed by prolonged wound healing complications (*n* = 2). Mechanical failure leading to amputation was rare and observed in only one patient with prosthetic loosening.

## 4. Discussion

The present study evaluated revision-free implant survival following MUTARS^®^ Total Knee reconstruction, with a particular focus on the comparison between primary implantation and use as a conversion procedure after failure of a previous knee prosthesis. The principal findings were twofold: first, revision-free survival declined substantially over time, reflecting the considerable complication burden associated with megaprosthetic reconstruction, and second, no statistically significant difference in implant survival was observed between primary implantation and conversion procedures.

The overall revision-free survival observed in this cohort was limited, with survival rates of approximately 70% at 1 year, 52% at 2 years, and 29% at 5 years. These findings are consistent with the known challenges of megaprosthetic knee reconstruction, although direct comparison with previously published studies must be interpreted cautiously [23,24,25]. Reported survival rates in the literature vary widely, typically ranging between 50% and 80% at 5 years, depending on patient selection, surgical indication, and endpoint definition [11,17]. The comparatively lower survival observed in the present study likely reflects the inclusion of a heterogeneous patient population comprising both extensive oncologic resections and complex revision cases with compromised bone stock and soft tissues.

A central objective of this study was to investigate whether implantation as a conversion procedure after failed arthroplasty is associated with inferior implant survival compared with primary megaprosthetic reconstruction. Conversion procedures are generally considered to represent a higher-risk clinical scenario, as these patients frequently present with multiple prior surgeries, altered soft-tissue envelopes, and an increased likelihood of previous or subclinical infection [13,22]. Interestingly, the present analysis did not demonstrate a statistically significant difference in revision-free survival between primary and conversion groups. Both Kaplan–Meier analysis and Cox regression failed to identify conversion procedures as a risk factor for revision.

This finding challenges the commonly held assumption that conversion procedures inherently lead to inferior outcomes [4]. However, several aspects must be considered when interpreting this result. First, the absence of a statistically significant difference does not imply equivalence between groups. Given the relatively small sample size, the study may have been underpowered to detect clinically meaningful differences, raising the possibility of a type II error. Second, visual inspection of the survival curves suggested a tendency toward earlier failure in the primary implantation group, particularly within the first two years after implantation. However, these differences did not translate into a statistically significant difference in revision-free survival between groups. Third, differences in follow-up duration between groups may have influenced survival estimates, as longer follow-up in the primary group increases the likelihood of observing late failures.

Failure mode analysis demonstrated that structural complications and periprosthetic infection were the predominant causes of revision. This distribution is consistent with previous reports on tumor endoprostheses, in which mechanical failure and infection are the leading causes of implant failure [4,26]. A more detailed analysis of structural failure revealed that component fracture and periprosthetic fracture were the most frequent mechanisms. This finding underscores the mechanical challenges associated with megaprosthetic reconstruction, particularly in patients with compromised bone stock and high biomechanical stress [27,28]. In contrast, mechanical loosening and disconnection were less common. The relatively low incidence of soft-tissue failure in the present cohort may reflect the frequent use of muscle flap reconstruction and other soft-tissue optimization strategies.

An important observation of this study is the marked variability in survival estimates depending on the definition of failure. When structural failure or infection were analyzed as isolated endpoints, survival rates differed substantially from overall revision-free survival. This finding highlights the importance of clearly defined and standardized endpoints in studies of megaprosthetic reconstruction, as different definitions of failure may lead to significantly different interpretations of implant performance.

The multivariable Cox regression and the additional Firth-corrected Cox regression analysis did not identify independent predictors of revision. While this finding may suggest that commonly assumed risk factors such as radiotherapy, prior infection, or implant coating do not independently influence revision risk, it is more likely that the analysis was limited by insufficient statistical power. The relatively small cohort size and the inclusion of multiple covariates increase the risk of overfitting and limit the ability to detect significant associations. The wide confidence intervals observed for several variables further support this interpretation.

The observed amputation rate of 19.4% highlights the severity of the underlying clinical scenarios treated with MUTARS Total Knee reconstruction. In 6 of 7 cases (86%), limb loss was associated with persistent infection or wound healing problems, whereas purely mechanical failure accounted for only a single case. This highlights that biological factors, rather than implant-related mechanical failure, represent the primary reason leading to amputation. The higher proportion of amputations in the primary implantation group likely reflects the oncologic complexity of these cases, including extensive resections, soft-tissue deficits, and infection risk. Importantly, most amputations occurred within the early postoperative period, suggesting that biological complications rather than late mechanical failure were the dominant drivers of limb loss.

Several limitations of this study should be acknowledged. The retrospective design introduces the potential for selection bias and limits control over confounding variables. The relatively small sample size reduces statistical power and may obscure clinically relevant differences between groups. The heterogeneity of the study population, including both oncologic and non-oncologic indications, complicates direct comparison with more homogeneous cohorts reported in the literature. Differences in follow-up duration between groups may have influenced survival estimates and should be considered when interpreting the results. Furthermore, functional outcomes and patient-reported measures were not assessed, and the present analysis therefore focuses exclusively on revision-free survival rather than overall clinical success.

The application of Cox proportional hazards regression in small datasets is associated with potential bias and instability of hazard ratio estimates [29]. To address this limitation, a Firth-corrected Cox regression was performed, which yielded results consistent with the conventional analysis [30]. Nevertheless, residual confounding and limited statistical power remain inherent limitations of this study. Despite these constraints, the present study provides clinically relevant insights into failure mechanisms following megaprosthetic knee reconstruction, particularly through the use of endpoint-specific analyses and detailed characterization of complications. The findings suggest that megaprosthetic reconstruction represents a viable salvage option even in complex revision scenarios, without clear evidence of inferior implant survival compared with primary implantation. Given the high overall complication rates observed, careful patient selection, meticulous surgical technique, and optimized perioperative management remain essential to improve outcomes.

Future research should focus on larger, preferably multicenter cohorts with standardized reporting of endpoints and inclusion of functional outcomes. Prospective studies are needed to better define risk factors for failure and to develop strategies aimed at reducing complication rates in megaprosthetic knee reconstruction.

## 5. Conclusions

In conclusion, MUTARS^®^ Total Knee implantation is associated with relatively high revision rates and limited long-term revision-free survival, regardless of implantation indication. Conversion to a MUTARS^®^ Total Knee prosthesis after failure of a previous knee arthroplasty did not result in inferior revision-free survival compared with primary implantation. These findings support the use of MUTARS^®^ Total Knee systems as a salvage option in complex revision scenarios while highlighting the substantial revision burden inherent to megaprosthetic knee reconstruction.

## Figures and Tables

**Table 1:** Patient Demographics and Surgical Characteristics.

Variable	Overall (*n* = 36)	Primary MUTARS Total Knee (*n* = 24)	MUTARS Total Knee as Conversion (*n* = 12)
Age, years, mean ± SD	46.3 ± 19.8	48.5 ± 21.9	42.1 ± 14.8
Male sex, *n* (%)	22 (61.1)	14 (58.3)	8 (66.7)
Follow-up, months, median (IQR)	16 (3–44)	16 (3–47)	19.5 (5–32.8)
Radiotherapy, *n* (%)	5 (13.9)	5 (20.8)	0 (0)
Silver-coated prosthesis, *n* (%)	21 (58.3)	20 (83.3)	1 (8.3)
Muscle flap reconstruction, *n* (%)	22 (61.1)	15 (62.5)	7 (58.3)
Cemented fixation, *n* (%)	15 (41.7)	11 (45.8)	4 (33.3)

**Table 2:** Kaplan–Meier revision-free implant survival.

Time Point	Overall Survival (%)	Primary MUTARS Total Knee (%)	MUTARS Total Knee as Conversion (%)
12 months	70 (*n* = 25)	72 (*n* = 17)	67 (*n* = 8)
24 months	52 (*n* = 18)	54 (*n* = 12)	49 (*n* = 6)
60 months	29 (*n* = 7)	31 (*n* = 5)	26 (*n* = 2)
Median survival, months	27	30	22

**Table 3:** Failure Modes According to the Henderson Classification.

Henderson Failure Mode	Overall (*n* = 36)	Primary MUTARS Total Knee (*n* = 24)	MUTARS Total Knee as Conversion (*n* = 12)
Type I—Soft-tissue failure	1 (2.8%)	0 (0.0%)	1 (8.3%)
Type II—Aseptic loosening	4 (11.1%)	4 (16.7%)	0 (0.0%)
Type III—Structural failure	11 (30.6%)	8 (33.3%)	3 (25.0%)
Type IV—Infection	8 (22.2%)	6 (25.0%)	2 (16.7%)
Type V—Tumor progression	0 (0.0%)	0 (0.0%)	0 (0.0%)

**Table 4:** Cox proportional hazards regression for risk of revision.

Variable	Hazard Ratio (HR)	95% Confidence Interval	*p*-Value
Age (per SD increase)	1.16	0.68–1.95	0.59
BMI over 30	0.37	0.07–2.05	0.25
Nicotin	1.50	0.44–5.18	0.52
Conversion vs. primary implantation	0.56	0.20–1.60	0.28
Silver-coated prosthesis	1.33	0.49–3.62	0.57
Radiotherapy	1.60	0.42–6.03	0.49
Preinfection	2.27	0.20–25.83	0.51

## Data Availability

The data presented in this study are available on request from the corresponding author. The data are not publicly available due to legal, ethical and privacy issues.

## References

[1] Henderson E.R., Groundland J.S., Pala E., Dennis J.A., Wooten R., Cheong D., Windhager R., Kotz R.I., Mercuri M., Funovics P.T. (2011). Failure mode classification for tumor endoprostheses: Retrospective review of five institutions and a literature review. J. Bone Jt. Surg..

[2] Theil C., Schneider K.N., Gosheger G., Schmidt-Braekling T., Ackmann T., Dieckmann R., Frommer A., Klingebiel S., Schwarze J., Moellenbeck B. (2022). Revision TKA with a distal femoral replacement is at high risk of reinfection after two-stage exchange for periprosthetic knee joint infection. Knee Surg. Sports Traumatol. Arthrosc..

[3] Theil C., Schwarze J., Gosheger G., Moellenbeck B., Schneider K.N., Deventer N., Klingebiel S., Grammatopoulos G., Boettner F., Schmidt-Braekling T. (2022). Implant survival, clinical outcome and complications of megaprosthetic reconstructions following sarcoma resection. Cancers.

[4] Sevelda F., Waldstein W., Panotopoulos J., Stihsen C., Kaider A., Funovics P., Windhager R. (2017). Survival, failure modes and function of combined distal femur and proximal tibia reconstruction following tumor resection. Eur. J. Surg. Oncol..

[5] Zhang H.R. (2022). Application and development of megaprostheses in limb salvage for bone tumors around the knee joint. Cancer Control.

[6] Gosheger G., Gebert C., Ahrens H., Streitbuerger A., Winkelmann W., Hardes J. (2006). Endoprosthetic reconstruction in 250 patients with sarcoma. Clin. Orthop. Relat. Res..

[7] Jeys L.M., Kulkarni A., Grimer R.J., Carter S., Tillman R., Abudu A. (2008). Endoprosthetic reconstruction for the treatment of musculoskeletal tumors of the appendicular skeleton and pelvis. J. Bone Jt. Surg..

[8] Grimer R.J., Aydin B.K., Wafa H., Carter S.R., Jeys L., Abudu A., Parry M. (2016). Very long-term outcomes after endoprosthetic replacement. Bone Jt. J..

[9] Biau D., Faure F., Katsahian S., Jeanrot C., Tomeno B., Anract P. (2006). Survival of total knee replacement with a megaprosthesis after bone tumor resection. J. Bone Jt. Surg..

[10] Gkavardina A., Tsagozis P. (2014). Use of megaprostheses for reconstruction of large skeletal defects. Open Orthop. J..

[11] Hu Y.C., Lun D.X. (2012). Prosthesis reconstruction techniques in malignant tumors around the knee. Orthop. Surg..

[12] Melnic C.M., Lightsey H.M., Lozano-Calderón S.A., Heng M. (2021). Megaprostheses in Nononcologic Hip and Knee Revision Arthroplasty. J. Am. Acad. Orthop. Surg..

[13] Palumbo B.T., Henderson E.R., Groundland J.S., Cheong D., Pala E., Letson G.D., Ruggieri P. (2011). Advances in segmental endoprosthetic reconstruction. Cancer Control.

[14] Byttebier P., Dhont T., Pintelon S., Rajgopal A., Burssens A., Victor J. (2022). Comparison of Different Strategies in Revision Arthroplasty of the Knee with Severe Bone Loss: A Systematic Review and Meta-Analysis of Clinical Outcomes. J. Arthroplast..

[15] Rodríguez-Merchán E.C., Gómez-Cardero P., Encinas-Ullán C.A. (2021). Management of bone loss in revision total knee arthroplasty: Therapeutic options and results. EFORT Open Rev..

[16] Kurtz S., Ong K., Lau E., Mowat F., Halpern M. (2007). Projections of primary and revision hip and knee arthroplasty in the United States from 2005 to 2030. J. Bone Jt. Surg..

[17] Haidukewych G.J., Petrie J.R., Adigweme O. (2014). The multiply-operated total knee replacement patient: Salvage options. Bone Jt. J..

[18] Carender C.N., Bothun C.E., Taunton M.J., Perry K.I., Bedard N.A., Pagnano M.W., Abdel M.P. (2024). 3D-Printed Metaphyseal Cones in Revision Total Knee Arthroplasties: Excellent Survivorship of 740 Cones at 5 Years. J. Bone Jt. Surg..

[19] Hardes J., Henrichs M.P., Gosheger G., Gebert C., Höll S., Dieckmann R., Hauschild G., Streitbürger A. (2013). Endoprosthetic replacement after extra-articular resection of bone and soft-tissue tumours around the knee. Bone Jt. J..

[20] Donner S., Gwinner C., Haffer H., Perka C., Kirschbaum S. (2026). Limb salvage in multiple revision total knee arthroplasty using customised implants: When sleeves and cones are no longer an option. J. Exp. Orthop..

[21] Pala E., Trovarelli G., Calabrò T., Angelini A., Abati C.N., Ruggieri P. (2015). Survival of modern knee tumor megaprostheses. Clin. Orthop. Relat. Res..

[22] Sculco P.K., Flevas D.A., Jerabek S.A., Jiranek W.A., Bostrom M.P., Haddad F.S., Fehring T.K., Gonzalez Della Valle A., Berry D.J., Brenneis M. (2024). Management of Bone Loss in Revision Total Knee Arthroplasty: An International Consensus Symposium. HSS J. Musculoskelet. J. Hosp. Spec. Surg..

[23] Medellin M.R., Fujiwara T., Clark R., Stevenson J.D., Parry M., Jeys L. (2019). Mechanisms of failure and survival of total femoral endoprosthetic replacements. Bone Jt. J..

[24] Toepfer A., Harrasser N., Schwarz P.R., Pohlig F., Lenze U., Mühlhofer H.M.L., Gerdesmeyer L., von Eisenhart-Rothe R., Suren C. (2017). Distal femoral replacement with the MML system: A single center experience with an average follow-up of 86 months. BMC Musculoskelet. Disord..

[25] Bernthal N.M., Greenberg M., Heberer K., Eckardt J.J., Fowler E.G. (2015). What are the functional outcomes of endoprosthestic reconstructions after tumor resection?. Clin. Orthop. Relat. Res..

[26] Mavrogenis A.F., Pala E., Angelini A., Calabro T., Romagnoli C., Romantini M., Drago G., Ruggieri P. (2015). Infected Prostheses after Lower-Extremity Bone Tumor Resection: Clinical Outcomes of 100 Patients. Surg. Infect..

[27] Haijie L., Dasen L., Tao J., Yi Y., Xiaodong T., Wei G. (2018). Implant Survival and Complication Profiles of Endoprostheses for Treating Tumor Around the Knee in Adults: A Systematic Review of the Literature Over the Past 30 Years. J. Arthroplast..

[28] Myers G.J., Abudu A.T., Carter S.R., Tillman R.M., Grimer R.J. (2007). The long-term results of endoprosthetic replacement of the proximal tibia for bone tumours. J. Bone Jt. Surg. Br. Vol..

[29] Jóźwiak K., Nguyen V.H., Sollfrank L., Linn S.C., Hauptmann M. (2024). Cox proportional hazards regression in small studies. Sci. Rep..

[30] Jiang N., Wu Y., Li C. (2024). Limitations of using COX proportional hazards model in cardiovascular research. Cardiovasc. Diabetol..

